# Evaluating the Feasibility, Acceptability, and Utility of the Home Alone Intervention: A Mixed Methods Pilot Study

**DOI:** 10.1155/jare/4036735

**Published:** 2026-05-19

**Authors:** Robyn W. Birkeland, Stephanie N. Ingvalson, Grace M. Savard, Amy Hobday, Dana Urbanski, Renée Pepin, Laura N. Gitlin, Jill Cigliana, Joseph E. Gaugler

**Affiliations:** ^1^ School of Public Health, University of Minnesota, Minneapolis, Minnesota, USA, umn.edu; ^2^ Department of Speech, Language and Hearing Sciences, Indiana University, Bloomington, Indiana, USA, indiana.edu; ^3^ Department of Community and Family Medicine, Geisel School of Medicine, Dartmouth University, Hanover, New Hampshire, USA, dartmouth.edu; ^4^ College of Nursing and Health Professions, Drexel University, Philadelphia, Pennsylvania, USA, drexel.edu; ^5^ Memory Care Home Solutions, Saint Louis, Missouri, USA

**Keywords:** home safety, mild cognitive impairment, solo living, subjective memory decline, support program

## Abstract

**Objectives:**

Loneliness and social isolation are associated with significant negative impacts on physical and mental health. Living alone increases one’s risk for loneliness and social isolation. Many older adults live alone, including individuals with mild cognitive impairment (MCI) and subjective memory decline. Living alone with a diagnosis of MCI or subjective memory decline adds greater complexity, stress, and difficulty in navigating daily life due to loneliness, social isolation, and negative mental and physical health outcomes. Despite these concerns, there is a dearth of support interventions for older adults who live alone with memory decline. Home Alone is a new support program created for this unique population of older adults in continuing to live independently and safely while engaging in social and meaningful activities. This pilot study evaluated the feasibility of the novel Home Alone support program.

**Methods:**

Home Alone is a seven‐session psychosocial and psychoeducational intervention that provides one‐on‐one, tailored coaching in person or via telehealth. Home Alone focuses on promoting home safety strategies and modifications and encouraging personally meaningful activity and social engagement through behavioral activation‐based tasks. Fifteen older adults who live alone and have MCI or subjective memory decline enrolled in the 3‐month feasibility study to evaluate the initial program and inform its refinement. Mixed methods data were collected from 1‐ and 3‐month follow‐up surveys. Additional qualitative data on participants’ experiences of the Home Alone intervention were collected at post‐intervention interviews.

**Results:**

Results demonstrated preliminary feasibility, utility, and acceptance of Home Alone. Qualitative analyses of participant interviews identified useful aspects of the intervention, including home safety, meaningful activity, and socialization, as well as recommendations for program refinement.

**Discussion:**

The Home Alone program was well‐received and viewed as valuable by participants. Participant feedback and recommendations were incorporated to further improve the intervention. The refined Home Alone intervention is now being evaluated in a larger pilot trial.

**Trial Registration:** ClinicalTrials.gov identifier: NCT05746390

## 1. Introduction

Loneliness and social isolation are common phenomena for older adults [1]. Loneliness is the subjective feeling of dissatisfaction with the quantity and quality of one’s relationships, while social isolation is an objective lack of a support network [2]. The experience of loneliness and/or social isolation is associated with a wide range of negative physical and mental health outcomes, including cardiovascular disease, mortality, depression, and decreased quality of life and cognitive function [1–3]. In fact, socially isolated individuals face health risks comparable to those of smokers and obese individuals [1].

Individuals with mild cognitive impairment (MCI), characterized as observable cognitive deficits that are greater than expected for a person’s age but do not significantly impact functioning [4], are at greater risk for experiencing social isolation and loneliness. This risk is exacerbated for individuals with MCI who live alone, a large and growing population. One in four Americans over the age of 55 who have cognitive impairment lives alone [5]. This number includes those with MCI [6]. Additionally, nearly 14% of adults aged 45 and older living alone in the United States report subjective cognitive decline [7]. Taken together, a sizable number of older adults who have some degree of memory concerns live alone.

Living alone in itself is associated with adverse health outcomes, including increased hospital admissions for respiratory disease and elevated mortality in older adulthood [8, 9]. In fact, older adults who live alone are at increased risk for cognitive impairment or dementia compared to those who do not live alone [10–14]. Unsurprisingly, individuals who live alone are also more likely to experience loneliness and social isolation [15].

Living independently in the presence of MCI or subjective memory decline adds complexity. Nearly half of older adults living alone with memory concerns or MCI struggle with at least one basic activity of daily living (ADL) or instrumental activity of daily living (IADL), and most do not receive assistance to address these challenges [6]. Individuals with MCI and subjective memory decline endorse greater levels of subjective stress, anxiety, depression, and difficulties in daily life [16–19], as well as reduced quality of life [18, 19]. People with MCI often experience challenges with driving safety and managing finances and medications [20], which present obstacles to social engagement and activity.

In contrast to the range of support designed for individuals with dementia, which typically incorporates family caregivers [21–23], there is a dearth of support interventions for older adults with MCI who live alone and often do not have caregiver support. To our knowledge, there has only been one feasibility‐tested intervention designed to support older adults living alone with MCI [24]. “Services To Age in Your Home” was noted to focus on increasing use of services, such as healthcare and social services, through 12 weekly in‐person sessions of a social worker‐guided care management intervention [24].

Given the desire many people have to age in place [25] and the rising number of individuals with MCI living alone [26], the lack of behavioral support programs for individuals living alone with MCI is a critical intervention gap. Evidence‐based programs that promote social engagement, activity, and safety are needed to help mitigate the profound health risks associated with isolation and cognitive decline. Furthermore, these programs can promote lifestyle strategies that may delay the need for significant assistance, ultimately leading to fewer transitions to residential care. To address this serious unmet need, the novel Home Alone support program was created.

Home Alone is a psychosocial and psychoeducational intervention designed specifically for older adults who live alone with MCI or subjective memory decline. This new intervention employs one‐on‐one tailored coaching conducted at home or via telehealth to support this unique population in continuing to live independently and safely while engaging in meaningful activities. Steeped in the theory of environmental press [27, 28], Home Alone strives to alleviate the demands and challenges imposed by one’s physical and social environment by reducing risk and confusion in the home through home safety strategies and organization and expanding problem‐solving and planning skills, particularly regarding social engagement and activity planning. To do so, Home Alone incorporates intervention elements from two evidence‐based programs: Skills2Care [29] and Brief Behavioral Activation for Improving Social Connectedness [30–32]. Home Alone follows the person‐centric approach featured in the Tailored Activity Program (TAP) [21, 33].

This pilot study employed a mixed‐methods approach to evaluate the appropriateness, acceptability, feasibility, and utility of the Home Alone intervention for older adults living alone with MCI or subjective memory decline and to gather feedback to adapt and refine the intervention for subsequent testing.

## 2. Methods

The Home Alone study was approved by the University of Minnesota Institutional Review Board (STUDY00017313). The study was registered in advance on ClinicalTrials.gov.

### 2.1. Intervention Design

The Home Alone intervention seeks to relieve environmental stress in this unique population by (1) decreasing home safety risks and sources of environmental confusion and (2) increasing engagement in values‐aligned activities. The intervention was designed in collaboration with an occupational therapist specializing in dementia supports (author JC), a geropsychologist with expertise in behavioral activation for social connectedness (author RP), and a sociologist/intervention scientist (author LG) who developed the evidence‐based dementia support interventions, Skills2Care [29] and TAP [21, 33].

Home Alone incorporated components of three well‐established mental health and dementia care intervention models: Skills2Care [29], which emphasizes home safety and organization, and behavioral activation [32, 34, 35], and TAP [21, 33], which emphasize engagement and personalized activity. These evidence‐based programs align with Home Alone’s purpose, as they aim to reduce sources of environmental stress [36] and promote engagement in personally meaningful activities to improve social well‐being and maintain cognitive function.

#### 2.1.1. Home Safety

Building on Skills2Care’s focus on home safety, the intervention includes a home safety audit to increase awareness of risks and reduce potential confusion in the home environment [29]. Home Alone also provides resources, psychoeducation, and support tailored to each participant’s individual needs and living circumstances.

#### 2.1.2. Behavioral Activation

To target socialization and activity engagement, we implemented key elements of Brief Behavioral Activation for Improving Social Connectedness [30–32], including psychoeducation, exploration of values, activity monitoring and planning, and intentional scheduling. In line with TAP’s person‐centric focus on identifying beneficial activities, participants strengthen their understanding of their personal values and then identify meaningful, values‐aligned activities to engage in.

Notably, social connectedness is a crucial element of the Home Alone intervention, which aims to address the increased risk of isolation and loneliness among older adults living alone [30–32]. Home Alone also works with participants to increase their awareness of existing social supports and promote the discovery of new social opportunities. Motivational strategies, goal‐setting, and varied approaches for managing negative thinking are incorporated to help minimize barriers and improve engagement in meaningful activities.

### 2.2. Intervention

Home Alone is a semistructured intervention comprising seven one‐on‐one psychoeducational coaching sessions delivered by an interventionist holding a doctoral degree in clinical psychology. Session discussions are tailored to address each participant’s individual concerns and needs regarding program content while adhering to the curriculum’s guidelines. Specific program content is detailed below (see Table 1). General agendas for each session are available in Supporting Item 1. Of note, these materials were used for piloting purposes, and the intervention has been modified and refined since this phase of development. Sessions 1–2 were semistructured, focused on home safety and resource provision. Sessions 3–7 focused on behavioral activation and thus were structured. Sessions were typically 45–60 min long and were held weekly, except for scheduling conflicts. Based on participants’ preferences, sessions were conducted by phone, via secure video conferencing, or in person (if participants lived within an hour’s drive of the University of Minnesota). Participants could use a mix of delivery modes based on their continuing availability and preferences during the study period. At any time during the intervention or after intervention completion, participants could request additional ad hoc sessions or email communications. This ad hoc support was designed to address emerging participant issues or concerns by providing resources and/or facilitating collaborative problem‐solving outside of regularly scheduled coaching sessions.

**TABLE 1:** Home Alone session content.

	Session focus/content	Session objectives
Session 1	• Build rapport • Gain understanding of participants’ perspectives/priorities and concerns • Pinpoint environmental aspects that may contribute to safety concerns or behavioral challenges • Set 1–2 activity goals	Interview, home safety audit, and relationship building

Session 2	• Continue to build rapport and trust • Discuss relevant environmental modifications (e.g., grab bar installation, decluttering) • Share tailored informal and formal supports and services • Share relaxation strategies, as time allows	Increase awareness and utilization of formal and informal long‐term services and supports

Session 3	• Provide rationale for behavioral activation • Explain the purpose of and demonstrate how to complete activity monitoring logs	Discover activities associated with positive mood

Session 4	• Explore life areas and participant values • Identify activities that reflect personal values • Identify values‐aligned activities to complete for homework	Values identification and determination of activities based on personal values

Session 5	• Educate on how environment and activity plans help with mood, perceptions of loneliness, and function • Explain the purpose of and demonstrate how to complete intentional scheduling logs	Intentional scheduling of activities associated with positive mood

Session 6	• Share strategies for engaging in activities and social situations, including positive self‐talk, motivational tips, SMART goal‐setting, and problem‐solving techniques. • Troubleshoot issues • Update intentional scheduling of activities for the week	Prepare for future considerations and obstacles

Session 7	• Review intervention concepts • Discuss any remaining concerns • Encourage continuation of skills learned and activities initiated as well as being open to engage in new activities	Review information Shared

Ad Hoc	• Resource provision • Timely problem‐solving	Address the needs of participants in between sessions or after intervention completion, if requested

Prior to the study’s start, the coach (corresponding author RB) completed 20 hours of behavioral activation training with the author, RP, and continued to meet via Zoom for approximately 45 min biweekly for ongoing consultation and fidelity monitoring (Months 1–24). The coach also completed 10 additional hours of environmental assessment training with the author, JC, to ensure appropriate delivery of the Home Alone Intervention.

The participant workbook was also designed in collaboration with author RP to include materials for participants to review in support of behavioral activation‐related learning occurring in session (e.g., information about the purpose of behavioral activation, values exploration and identification, and motivational strategies). Daily living strategies, including SMART goal‐setting, were also shared as a resource in the workbook. Additionally, the workbook included activity logs and intentional scheduling logs for participants to complete as homework, based on Lejuez et al.’s (2011) *Brief Behavioral Activation Treatment for Depression: Revised Treatment Manual* [35]. The home safety audit was created by adapting items from standard home safety checklists available on the AARP and Alzheimer’s Association websites to apply to individuals living alone with memory impairment, while incorporating recommendations from author JC, an occupational therapist. The audit was conducted as a semistructured interview tailored to each individual’s home environment. Additionally, the coach visually inspected participants’ homes for safety risks during in‐home visits. Other participants either showed their home as best they could via video conferencing or provided descriptions. All participants were referred to their websites to view the publicly available home safety checklists and resources. Session notes included sections suggested by author RP to properly document behavioral activation‐related session tasks.

### 2.3. Recruitment

Recruitment took place from May 2023 to November 2023. Participants were recruited via informal and formal dementia caregiver registries, newspaper articles, educational events, social media platforms, the Home Alone study website, partnerships with local memory clinics, and word‐of‐mouth communication. Additionally, the study team promoted Home Alone through outreach with community aging organizations and independent living communities.

### 2.4. Eligibility Criteria

Participants were at least 55 years old and living alone in the United States. Participants were eligible if they met one or more of the following criteria: (1) had a provider diagnosis of MCI or other cognitive impairment; (2) scored between 13 and 18 out of 22 points, indicative of MCI [37], on the Blind/Telephone Montreal Cognitive Assessment (B/T‐MoCA) administered during a screening call [38]; or (3) self‐identified with subjective memory decline. Notably, the initial inclusion criteria did not include self‐identification of subjective memory decline. This criterion was added in mid‐May 2023 after our first wave of recruitment brought many individuals with subjective memory decline requesting help, but no one diagnosed with MCI had been reached. Therefore, a decision was made to allow individuals with subjective memory decline to enroll to meet their needs and facilitate recruitment for the study. Individuals were excluded from the study if they: (1) lived in an assisted living community, group care home, or similar residential setting that provided care and services; (2) scored 12 or less on the B/T‐MoCA administered by study staff at the time of screening; (3) did not speak English; (4) were participating in a coaching or counseling program specific to independent living; (5) had a new or worsening mental health condition and were not receiving ongoing treatment; (6) had a change in dosage of psychotropic medications in the last three months; (7) were unable to pass an adapted University of California, San Diego Brief Assessment of Capacity to Consent (UBACC) at the time of consent [39]; or (8) were not willing/interested/able to participate actively in the intervention, per researcher discretion.

**FIGURE 1:**
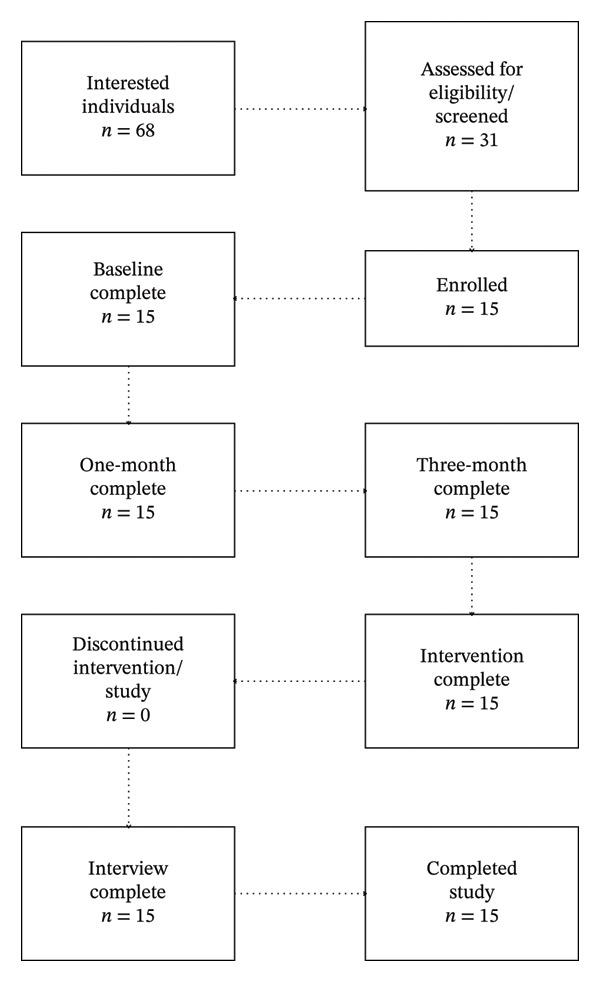
Participant flow.

Study staff established contact with 68 interested individuals, and 31 were formally screened. Fifteen of those screened consented and enrolled, an appropriate *n* for a pilot feasibility study [40, 41]. Participants who were formally screened but did not fully consent and enroll either did not meet eligibility criteria during the screening process (*n* = 12) or were lost to follow‐up (*n* = 4). Participants provided verbal consent by phone after passing the UBACC. Ineligible individuals were offered resources, including the Alzheimer’s Association website and 24/7 helpline, contact information for their Area Agency on Aging, and individualized resources to address specific concerns. See Figure 1 for participant flow.

### 2.5. Measures

This study employed a convergent parallel mixed‐methods approach to evaluate the feasibility, appropriateness, acceptability, and utility of the Home Alone intervention and to refine intervention elements (session number, length, and mode of delivery) for future study phases.

#### 2.5.1. Evaluating Administrative Feasibility: Recruitment, Retention, and Fidelity

Study staff utilized secure contact logs to track recruitment efforts and timelines for participant correspondence. The research team met regularly to discuss progress, ensure accountability, and maximize coordination efforts.

The Home Alone coach maintained a contact log with session dates for all participants. The coach also kept individual contact logs for each participant, documenting the date of each communication, its length and mode, session topics, and purpose. After each session, the coach also completed notes reflecting the key topics discussed, activities engaged in, homework assigned, and plans for the next session. These records served to promote and track treatment fidelity in conjunction with biweekly supervision and review with author RP.

#### 2.5.2. Acceptability, Feasibility, Appropriateness, and Utility of the Intervention

In addition to participant retention and intervention completion, we also evaluated the acceptability, feasibility, appropriateness, and utility of the intervention, assessing these domains in follow‐up surveys at one and 3 months, as well as a semistructured interview at the end of each participant’s study period.

#### 2.5.3. Survey and Interview Administration

Study staff administered the baseline survey to participants via telephone (*n* = 14) and mail (*n* = 1). In addition to collecting sociodemographic information, specific measures were selected to evaluate social interactions and networks; sensory impairment and aids; cognitive impairment; help or assistance with activities of daily living; utilization of unpaid and paid support services; participation in meaningful activities; physical activity; and emotional health. Of note, these measures were included in this phase solely to assess their feasibility and acceptability with this population to inform their fit for inclusion in a subsequent phase II evaluation. Detailed descriptions of the survey measures and their references are available in Supporting Item 2.

Follow‐up surveys were administered approximately 1 and 3 months after the baseline survey was completed. Follow‐up surveys included the baseline measures, except for sociodemographic items. As this pilot study was focused only on feasibility, appropriateness, acceptability, and utility of the novel Home Alone intervention, the follow‐up surveys included three brief measures assessing the acceptability, appropriateness, and feasibility of the intervention: the acceptability of intervention measure (AIM), the intervention appropriateness measure (IAM), and the feasibility of intervention measure (FIM), respectively [42]. Each measure consists of four items, each with a 5‐point Likert scale (1 = *completely disagree*, 5 = *completely agree*). All three measures have demonstrated validity, with high internal consistency (AIM *α* = 0.85, IAM *α* = 0.91, and FIM *α* = 0.89) and high test‐retest reliability (AIM = 0.80, IAM = 0.73, FIM = 0.88). The fourth measure, a 10‐item treatment receipt checklist (TRC), was adapted for this study from other dementia care interventions delivered by the research team [43, 44]. The TRC used a 5‐point Likert scale (1 = *strongly disagree* to 5 = *strongly agree*, with an option to indicate “not applicable to me/my situation”). Nine items assess receipt of intervention content (such as discovery of personal values, scheduling activities, home modifications, and goal‐setting) and acceptability, utility, and inclination to recommend the intervention to others in similar situations. The TRC also included an open‐ended question seeking participants’ feedback on whether the intervention was helpful to them.

Study staff administered the first follow‐up survey via telephone (*n* = 14) and mail (*n* = 1), and the second follow‐up survey by telephone (*n* = 9), mail (*n* = 3), or electronically via Qualtrics, a secure online survey platform (*n* = 3). In addition to mail or phone completion, surveys were also offered online via Qualtrics beginning in January 2024, based on participant feedback that completing over the phone with study staff or via mail took too much time and was burdensome, particularly after having to complete the consent process, UBACC, and B/T‐MoCA before each assessment time point. This new online method of survey completion was offered late in the study to determine whether it would be a feasible approach for Phase II.

The average duration for survey completion of the 1‐month survey, including the outcome measures, was 68 min (SD = 10.06, range 46–80 min, *n* = 14, as one survey was completed by mail). For the 3‐month survey, also including outcome measures, the average duration of survey completion was 53 min (SD = 22.59, range 20–85 min, *n* = 12, as two surveys were completed by mail and the recorded timing for one was an error). The duration of Qualtrics surveys completed directly by the participant (*n* = 3) was considerably shorter (range: 20–41 min) than that of surveys completed with study staff over the phone. Since the implementation measures (AIM/IAM/FIM/TRC) were included in the full survey with the piloting of the outcome measures, it is unclear how long they took to complete. However, it is likely that they were completed in approximately 5–10 min, as the measures are short and the items are not complicated.

After completing the intervention, participants engaged in an individual audio‐recorded, semistructured interview via phone or video conference with a graduate research assistant (author GS) to share their experiences of the Home Alone study. The interview focused on participant impressions; target outcomes (i.e., participation in meaningful activities, socialization, impacts on mood and health, awareness of or utilization of supports and services, confidence in living alone, and home safety modifications); current needs; program delivery; and future recommendations. The 19‐question interview guide is available in Supporting Item 3. The study ended after the 15 participants had completed the intervention, all follow‐up surveys, and the interview.

### 2.6. Data Analysis

#### 2.6.1. Quantitative

The data from the three surveys (baseline, 1‐month, and 3‐month follow‐up) were collected using Qualtrics and downloaded to a secure server upon completion of the study. The data were cleaned and compiled on SPSS. Frequency tables and means were created to summarize participant demographics (Table 2). For the means and SDs of sum scores of the behavioral, cognitive, and health measures, included in the assessment to evaluate the feasibility of using those measures for future RCT evaluation, see Supporting Item 4. Data analysis included all enrolled participants and focused solely on empirical descriptions of feasibility, appropriateness, and acceptability. Tables for frequencies and means for the FIM, AIM, and IAM measures (Table 3) and item‐level responses for the TRC (Table 4) were also created. The percentage of participants who selected either *agree* or *strongly agree* (percent agreement) for each item in the feasibility, acceptability, and appropriateness measures (FIM/AIM/IAM) at the 1‐ and 3‐month surveys was also calculated. The few “not applicable to me/my situation” responses were not included in the calculations. See Table 4 for additional details.

**TABLE 2:** Demographics.

	*N*	% (SD)
	15	100
Age (mean)	73.33	(8.59)
Diagnosis of cognitive impairment	6	40
Female	11	73.3
White	14	93.3
Married	0	0
Number of living children (mean)	2.5	(1.7)
Education: bachelors or higher	13	86.7
Employed	1	6.7
Household income 40K+	7	46.7
N (of those that have children)	11	73.3
Talk with children 1/week or more	9	81.8
Visit with children 1/week or more	5	45.5

**TABLE 3:** Implementation outcomes: intervention acceptability, appropriateness, and feasibility.

	T1		T2	
	Mean (SD) range	*Strongly agree* or *agree* (%)	Mean (SD) range	*Strongly agree* or *agree* (%)
*Acceptability of intervention (AIM)*				
1. Home Alone meets my approval.	4.47 (0.516) 4–5	100	4.47 (0.516) 4–5	100
2. Home Alone is appealing to me.	4.33 (0.617) 3–5	86.7	4.47 (0.640) 3–5	93.3
3. I like Home Alone.	4.33 (0.724) 3–5	80	4.47 (0.516) 4–5	100
4. I welcome Home Alone.	4.20 (0.775) 3–5	80	4.40 (0.632) 3–5	93.3

*Intervention appropriateness (IAM)*				
1. Home Alone seems fitting.	4.07 (0.884) 2–5	80	4.07 (0.799) 2–5	86.7
2. Home Alone seems suitable.	4.13 (0.834) 2–5	86.7	4.20 (0.561) 3–5	93.3
3. Home Alone seems applicable.	4.07 (0.799) 2–5	86.7	4.20 (0.414) 4–5	100
4. Home Alone seems like a good match.	4.07 (0.884) 2–5	80	3.93 (0.799) 2–5	80

*Feasibility of intervention (FIM)*				
1. Home Alone seems implementable.	4.00 (0.535) 3–5	86.7	4.13 (0.640) 3–5	86.7
2. Home Alone seems possible.	4.13 (0.516) 3–5	93.3	4.20 (0.561) 3–5	93.3
3. Home Alone seems doable.	4.13 (0.640) 3–5	86.7	4.27 (0.458) 4–5	100
4. Home Alone seems easy to use.	4.07 (0.594) 3–5	86.7	4.13 (0.743) 2–5	93.3

*Note:* All 15 participants completed each item at both timepoints.

**TABLE 4:** Treatment receipt checklist outcomes.

Home Alone helped me …	T1		T2	
	Mean (SD) range	*Strongly agree* or *agree* (%)	Mean (SD) range	*Strongly agree* or *agree* (%)
1. Discover what activities make me happy.	3.73 (0.704) 3–5	60	3.73 (0.704) 3–5	60

2. Make changes around my home to make things easier for me.	3.60 (0.828) 2–5	53.3	3.64 (1.008) 2–5	57.1

3. Learn how to do the things I enjoy.	3.43 (0.852) 2–5	35.7	3.38 (1.044) 1–5	38.5

4. Explore my values.	3.75 (0.622) 3–5	66.7	4.13 (0.640) 3–5	86.7

5. Schedule activities.	3.53 (0.834) 2–5	46.7	3.80 (0.775) 2–5	73.3

6. Learn how to set goals.	3.8 (0.862) 3–5	66.7	3.93 (0.829) 2–5	78.6

7. Problem‐solve challenges to being active and social.	3.69 (0.855) 3–5	46.2	3.62 (0.768) 2–5	61.5

8. The Home Alone intervention was useful to me.	4.00 (0.845) 2–5	80	4.07 (0.458) 3–5	93.3

9. The Home Alone intervention was acceptable to me.	4.40 (0.507) 4–5	100	4.33 (0.488) 4–5	100

10. I would recommend Home Alone to others in a similar situation as I am.	4.27 (0.704) 3–5	86.7	4.33 (0.617) 3–5	93.3

*Note: N* = 15 at both time points. Scores on a scale from 1 (*strongly disagree*) to 5 (*strongly agree*), with an option for not applicable (N/A). For the 1‐month survey, one participant selected N/A for item 3, three participants for item 4, and two participants for item 7. For the 3‐month survey, one participant selected N/A for item 2, two participants for item 3, one participant for item 6, and two participants for item 7.

#### 2.6.2. Qualitative

Transcripts (*n* = 15) of the semistructured interviews and the open‐ended item responses from the study surveys were qualitatively analyzed by the analysis team (authors SI, RB, GS, AH). Thematic analysis was conducted using Braun and Clarke’s framework [45]. A detailed record documenting the qualitative research plan, including the review process and weekly transcript assignments, was kept. The analysis team independently read two different and randomly assigned transcripts to generate initial codes. Following independent code generation, the team held an in‐depth group discussion on code relevance and drafted an initial codebook. Team members independently coded additional transcripts using the initial codebook and met subsequently to review transcripts, revise the codebook for accuracy, and add detailed descriptions for each code. Team members met after each round of coding. Discrepancies were discussed and resolved as a group at each meeting. After six rounds of preliminary coding, saturation was met, and the codebook was finalized. The final codebook includes 25 codes: 17 codes related to the perceived impacts of the intervention, five codes related to the intervention structure and delivery, two codes regarding participants’ recommendations and suggested modifications, and one code regarding participants’ concerns for their future (see Supporting Item 5). Each transcript was then coded individually on NVivo 14 by three coders (authors SI, RB, and AH) to allow for investigator triangulation and to evaluate inter‐rater reliability. Supporting Item 6 illustrates the qualitative analysis process.

## 3. Results

### 3.1. Sample Characteristics

The average age of participants was 73.3 years (range: 55–88 years, SD = 8.59). Forty percent of the participants had a diagnosis of some type of cognitive impairment, while the rest self‐reported subjective cognitive decline or had scored within the range suggestive of MCI on the B/T‐MoCA screening procedures. The average B/T‐MoCA score was 18.81 (range: 14–22, SD = 2.55). Twelve participants lived in the Midwest (67%), two in the West, and one in the South. See Table 2 for additional demographics. Demographics did not appear to vary by cognitive status (MCI diagnosis, B/T‐MoCA score within the MCI range, and subjective cognitive decline). See Supporting Item 7 for the table of demographics by cognitive status.

Every participant completed the seven‐session intervention. There were no differences in engagement with the intervention, regardless of the delivery method. In addition, most engaged in ad hoc support, primarily via email, which frequently involved resource provision. None of the participants opted to engage in an ad hoc session. There were no significant or unexpected adverse events. There were some adverse events that could be expected, given the age of the study sample, including hip replacement surgery, falls (no significant injuries), and gastrointestinal issues. Session and ad hoc communication data are available in Supporting Item 8.

### 3.2. Implementation Outcomes

All participants completed the intervention, indicating strong feasibility and acceptability. Results from the three implementation measures, assessing participant ratings of intervention acceptability (AIM), feasibility (FIM), and appropriateness (IAM), administered at 1 month and 3 months, also revealed positive perceptions of the intervention (see Table 3). Each participant (*N* = 15) completed all items for each implementation measure at both time points. At the 1‐month survey (administered prior to intervention completion), the average scores for acceptability, feasibility, and appropriateness were 4.33 (SD = 0.61), 4.08 (SD = 0.50), and 4.08 (SD = 0.83), respectively, with 5 representing the highest possible score.

Mean scores were higher at the 3‐month survey once the intervention was complete: acceptability *M* = 4.45 (SD = 0.54), feasibility *M* = 4.10 (SD = 0.54), and appropriateness *M* = 4.18 (SD = 0.57). At both time points across the three measures, most individual items demonstrated high agreement (indicated by responses of *agree* and *strongly disagree*). Additionally, at both time points, all 15 participants approved the intervention, and no individual item received less than 80% agreement.

Each participant also completed all items on the TRC at 1 and 3 months as well. Similarly, participants showed high levels of agreement on the TRC (see Table 4), with a higher percentage of agreement at the 3‐month follow‐up compared to the 1‐month administration. At 3 months, the majority of participants agreed or strongly agreed with most questions, indicating that they valued the intervention content, found the program useful, and would recommend Home Alone to others in a similar situation. Notably, at both time points, all participants agreed or strongly agreed with the item, “The Home Alone Intervention was acceptable to me.” In contrast, approximately two‐thirds of participants disagreed that “Home Alone helped me learn how to do things I enjoy” at both follow‐up surveys. At 3 months, this was the only survey item with less than 50% agreement among participants. This discrepancy is likely the result of a poorly worded item. Home Alone did not teach participants how to do activities they would enjoy, as one might have believed the item was asking. A better‐worded item would read, “Home Alone helped me identify activities I enjoy or find meaningful.”

### 3.3. Qualitative Results

Themes from the participant interviews identified aspects of the intervention that participants either found appropriate or useful, particularly in home safety, meaningful activity, socialization, daily living skills, motivational strategies, resource provision, and mood. Exemplar quotes for each theme are listed in Table 5. Additionally, participants shared recommendations for refining the intervention. Combined, the identified themes and recommendations will serve as guides for determining which program aspects to maintain and which to change for the next phase of evaluation. The average coding agreement was 96.6%. To protect anonymity, participants are referred to by pseudonyms.

**TABLE 5:** Theme exemplar quotes.

Participant (age)	Quote
**Home safety**	
*Home safety—implementation*	
Laura (65 yo)	“I picked up the ‘I’ll trip on it’ rugs.”
Sandi (72 yo)	“I kind of rearranged some areas that I used constantly because it was cluttered. I needed to organize.”
Marsha (55 yo)	noted how the intervention led her to install a grab bar in her bathroom, explaining, “I’m a fall risk [standing] in my bathtub.”

*Home safety—planning*	
Jessica (80 yo)	“I got a new handrail to put on the lower half of my stairs. I haven’t put it up yet.”
Eloise (86 yo)	“For safety, [my coach] suggested that I get one of those flexible shower heads, so I plan on doing that.”

**Meaningful activity**	
*Values identification*	
Phyllis (68 yo)	“I thought it was very useful. It gave me kind of a totally different framework to look at the stuff I do, what has value, what has meaning to me, and the things I do, and what I find pleasure in doing. So yeah, I thought it was awesome. In particular, the tools for figuring out…what brings meaning to me, what’s fun at the same time.”
Jessica (80 yo)	“…it has made me step back and take a look at what I have been doing or, more importantly, what I have not been doing to make my ability to stay in my house and live alone successfully. And a lot of the things like the [Values Identification] exercises have pointed out places where I could easily…make some modifications to how I’m living my life that will enrich it and make it better for me…and anything you can do to improve your quality of life is a good thing in my estimation.”

*Importance and identification of meaningful activities*	
Rich (74 yo)	“I think it’s where you set up— it’s like a chart or a spreadsheet of activities that add value to your life. What are they? Well, first of all [for me], it’s your family, it’s your faith, it’s basically what are the activities you do like, which ones you don’t like, but have to be done every day? That’s, I think, the most important thing I got from it.” He continues to use the values‐aligned activity list daily, “I took [the Home Alone] spreadsheet, and I use it also on my calendar and my 3 × 3 post‐it notes.”
Jessica (80 yo)	“I am not doing any more activities than I did before, but I have identified some areas that, especially through the cold winter months, will be indoor activities that I will begin to participate in…I sort of knew that the [local] senior center had a lot of activities going on, but I’d never really looked at them, but Home Alone pointed some of them out to me, and so now I have looked more deeply into what they offer and pinpointed some of the ones that I think I might be interested in participating in.”

*Implementation of meaningful activity*	
Jenny (79 yo)	“It encouraged me to take more initiative to do fun activities…That is a valuable skill, not waiting for someone else to organize things, but to get going.”
Dan (66 yo)	“I live at an over 55 apartment complex, and they have activities in there too. I started doing things.”
Phyllis (68 yo)	“I went and tried out a couple of different pinochle groups in the area. And I also volunteered at a new place, a different food pantry in town.”

*Intentional scheduling*	
Margaret (68 yo)	It reminded me to schedule some fun activities, sharing that she started to do things “…out in nature. That’s very useful to me, and I went to the movies…which I haven’t done in, I don’t even know how long…” She added that she started talking to someone at the movies and invited him to lunch, relating, “so that was a reminder to do some fun things, and then something else fun came out of that.”

*Did not impact activity level*	
Phil (80 yo)	“…I do have some plans for some fun activities for the future…I don’t want to overload myself. I have a lot of activities.”
Mary (88 yo)	felt that Home Alone’s focus on activity engagement “wasn’t particularly useful…I think that it was aimed at trying to get people to be more active, and I’m already active.”

**Socialization**	
*Impact on socialization*	
Jenny (79 yo)	“It helps to make it clear … the importance of getting with other people and being social and staying in contact so that when the opportunity arises to join an activity or be with other people, you’re more encouraged to do that, to be with other people.” “…there’s one particular relationship I have now with another woman….we got to know each other, and then normally I probably wouldn’t have really tried to stick with it or get to know her more. I would just let whatever happen, happen. But I was more forthcoming with her, and I invited her to do things, and she invited me to do things, and now, we’re best buds or whatever.”
Jessica (80 yo)	“I’m seeking other new activities that will…maybe overcome my natural inclination to be a hermit…If I want to successfully continue to live alone, I have to work on overcoming that habit that has built up badly since COVID.”

*Did not impact socialization*	
Eloise (86 yo)	“I actually think I spent quite a bit of time with other people already.”

**Implementation of daily living skills and motivational strategies**	
Beth (76 yo)	When asked to share the most useful part of the program, Beth stated, “making goals. I made a goal to go to an activity…I went today, and I intend to go tomorrow.”
Sandi (72 yo)	Sandi appreciated the tips on “what to improve on and having some structure in my day,” further elaborating that routine and organization were sometimes challenging to establish on her own.

**Initiation of services and supports**	
Marsha (55 yo)	shared that her coach “sent me some kind of link that connected me to updated programs, and I found a senior companion through Catholic Social Services.” She continued, “It’s a lifesaver to know about these things, because it’s not just me. I’ve been able to share it with other people.”
Laura (65 yo)	“I know what supports are out there…but to get it done for a person with my situation—I guess I need another human being to actually help me do it.”
Mary (88 yo)	received a senior housing guide from her coach after talking “a little bit about housing options as I aged… and that, in some ways, was helpful.” She added that, “I’ve gone with my daughter‐in‐law, and we’ve toured a couple of the places, which was informative, the senior living ones, so [I have] a feel for what they are.”

**Impact on mood and health**	
*Impact on mood*	
Phyllis (68 yo)	“I always felt better after I had talked to [my coach], and I do feel better when I get out and do stuff. So yeah, I think it did improve my mood.”
Dan (66 yo)	“It definitely improved my mood…What I liked [was the coach] wasn’t real pushy or anything, and it just made my mood better overall.”
Richard (74 yo)	“It taught me one thing, because every day you go up and down in your moods. It’s just normal. But it taught me to not let those moods, negative ones, affect me all the time.”

*Impact on health*	
Jessica (80 yo)	“It has focused my attention on the things that I can and should be doing to—both from a physical health [perspective]—like going to the fitness center and working out, and seeing my counselor to help on the emotional side.”
Laura (65 yo)	Noted that affirmation from her Home Alone coach helped motivate her to visit the doctor to check her blood pressure, update prescriptions, and develop a healthy walking routine.
Jenny (79 yo)	“It encouraged me to be a little more proactive, I would say that it contributed to my deciding to reach out to some medical personnel about issues I was having rather than to just let them go.”

### 3.4. Home Safety

The importance of discussing home safety emerged as a key theme in participants’ reflections on the Home Alone intervention. Many participants described making concrete changes to their living spaces to make them safer due to Home Alone, such as installing grab bars in their bathrooms. Some participants found decluttering recommendations helpful. Others were actively planning to implement the home safety recommendations but had not followed through at the time of the interview. While these participants had not yet fully implemented the changes, they reflected that the program identified immediate needs and introduced practical solutions they planned to adopt in the future.

### 3.5. Meaningful Activity

Participant interview responses often centered on the importance of meaningful activities in the intervention, including identifying values, determining value‐aligned activities, increasing awareness of opportunities for meaningful activities, and engaging in meaningful activities.

#### 3.5.1. Values Identification

To define what constituted a personally meaningful activity, each participant engaged in a component of behavioral activation centered on value identification. Participants explored their core beliefs and sense of self to identify their unique values and subsequently determined activities that align with these values. Participants found this novel exercise valuable, particularly for helping them pinpoint and reflect on what matters to them. They connected the idea that exploring their values and identifying activities that reflect those values could improve their quality of life and help maintain their ability to live independently.

#### 3.5.2. Importance and Identification of Meaningful Activities

Participants often described how Home Alone highlighted the importance of identifying and engaging in meaningful activities. They noted that Home Alone helped them discover opportunities for meaningful engagement aligned with their identified values and interests.

#### 3.5.3. Implementation of Meaningful Activity

Many participants noted increased engagement in meaningful, values‐aligned activities during the study. These participants often described engaging in new activities or trying new approaches to an existing activity. Other participants described resuming meaningful activities they had previously put on hold. Participants also appreciated the program’s emphasis on intentionally scheduling pleasant activities into one’s daily routine.

Not all participants engaged in more activities. Those who did not increase their activity engagement reported that they were already engaged in enough activities and were hesitant to add more to their already full plate.

### 3.6. Socialization

Across interviews, it was evident that many participants found the coaching discussions heightened their awareness of the need for socialization, particularly to curb loneliness and isolation. Participants not only described increased awareness of the importance of socialization but also illustrated how they translated that awareness into greater social engagement.

### 3.7. Implementation of Daily Living Skills and Motivational Strategies

When discussing the daily living skills, memory aids, and motivational strategies shared during the intervention, participants named goal‐setting and organizational strategies as the most useful.

### 3.8. Initiation of Services and Supports

Participants appreciated that Home Alone provided personalized, local resources to help support them. The coach found resources for a range of services and supports tailored to each participant’s needs and requests. The provision and discussion of resources also encouraged participants to consider or initiate service use that they might not have otherwise.

### 3.9. Mood and Health

The majority of participants felt that Home Alone sessions may have affected their mood, often highlighting the impact of the session content and the conversational nature of coaching. Others specifically discussed how they acquired skills for practicing emotional regulation. Several participants also discussed how the Home Alone program provided tools to bolster their confidence and even promoted self‐advocacy.

Some participants reported a renewed, more targeted focus on healthy lifestyle choices and practices during the study. A few spoke about how the program motivated them to seek out medical care. On the other hand, some participants expressed that Home Alone did not affect their mood or health.

### 3.10. Participant Feedback for Phase II

The majority of participants found the number of sessions, session length, and mode of delivery options appropriate. Several participants appreciated how the workbook helped facilitate the adoption of lifestyle skills and strategies. These aspects will remain unchanged for subsequent testing in a Phase II study. However, participants suggested content they felt should be included in future iterations of the Home Alone intervention, including a more thorough discussion of food security and an exploration of wearable technology and its applications. Multiple participants suggested additional time to discuss more personal concerns. A few participants agreed that the intervention seemed to follow a set agenda, with little leeway to hold additional conversations.

## 4. Discussion

As noted in prior National Institute on Aging’s Dementia Care and Caregiving Summits, a critical gap in dementia care science is not only to describe the challenges that face people living alone with dementia but also to develop and evaluate rigorous support programs for such individuals [46, 47]. To our knowledge, there are no psychoeducational support interventions that provide much‐needed support to older adults living alone with MCI or subjective cognitive decline. To meet this need and contribute to the literature on this unique and growing population, the novel Home Alone intervention was created. The principal goal of the current study was to assess the feasibility of the Home Alone intervention (based on behavioral activation and environmental press principles) and to determine whether the intervention required refinement/adaptation for a subsequent Phase II pilot study.

Overall, participants found the content and structure of Home Alone acceptable, useful, and feasible, supporting the initiation of a Phase II pilot study with minor modifications. These results are notable and offer preliminary evidence that Home Alone may be a viable option to help fill a critical need for a unique population that currently lacks established support. The findings of feasibility and acceptability were evident in practice and indicated that overall, the intervention was designed at an appropriate cognitive level and required an acceptable amount of effort for participants. Participants thoughtfully engaged with the coach, understood the behavioral activation‐oriented program material, and initiated meaningful activities independently. For any programmatic elements they found challenging, they shared constructive feedback and offered recommendations for future modifications. Their suggested modifications were incorporated into the refined intervention for use in a Phase II study.

In regard to intervention utility, the majority of participants reported that Home Alone’s Behavioral Activation components emphasized the importance of engaging in activities and helped them discover activities that brought them enjoyment. These findings align with other studies that have found behavioral activation increases meaningful activity engagement [30, 31, 48].

A few participants felt they were already engaging in an appropriate number of activities or even too many. Similarly, multiple participants reported being satisfied with their pre‐intervention social activity levels and did not feel the need to change them in response to the program. This finding is in contrast with much of the existing literature that suggests that living alone, memory concerns, and/or being an older adult are all associated with loneliness and social isolation [1–3]. However, as Clare et al. posit, living alone does not *ipso facto* mean one experiences a lack of social support [49]. Based on this participant feedback, the subsequent phase of the Home Alone pilot testing places less emphasis on increasing activity and social engagement for all participants. Instead, the focus is on personalized goals for socialization and activity, including for those who are more socially involved, improving the quality of their social activities, or for those overwhelmed with activities, prioritizing and reducing less personally meaningful ones.

Although there exist few, if any, interventions to support people living alone with cognitive impairment to serve as comparators to Home Alone, the multilevel focus of Home Alone on both behavioral activation and preferred activities is in keeping with socioecological models of dementia care [50]. Much of the dementia care intervention literature has adopted an individual‐level focus (e.g., on either the person living with dementia or the caregiver), which has in part led to inconsistent findings of efficacy or effectiveness of many dementia care intervention types. However, those dementia care interventions that target multiple levels of potential influence on key outcomes (e.g., individual, caregiver/care recipient dyad; home/built environment) appear to hold promise in achieving health benefits for people living with dementia [51, 52]. In this regard, Home Alone represents a dementia care intervention that adopts a multilevel approach (person, environment) to achieve perceived benefit for people living alone with cognitive impairment.

This study has several limitations. The sample size, although appropriate for a pilot feasibility study, was small. The 3‐month follow‐up period limited our understanding of the intervention’s possible long‐term effects. Another limitation is that participants willing to join the study were more likely to be proactive, socially engaged, and higher‐functioning, which may limit generalizability to lower‐functioning individuals with MCI. Participants’ activity and social engagement were based on self‐report. Moreover, for three participants who participated by phone, the coach relied on their perspectives on the safety and condition of their home. There is a chance that participants exaggerated their level of activity or that self‐audits of home safety understated or underappreciated the home’s safety risks. Finally, there was also only one coach for the intervention. As such, it is possible that results of the study may be more strongly associated with the coach’s performance than the intervention.

The Phase II study is currently underway. Phase II, along with future testing, will continue to gather in‐depth participant feedback to inform ongoing refinement and adaptation of the intervention in alignment with the NIH Stage Model for behavioral intervention development [53–56]. In addition to continued refinement of program content and delivery, subsequent testing will identify and test the intervention’s mechanisms of action—including those that informed its development (behavioral activation and environmental fit principles)—as well as any unintended mechanisms. Results will be used to adjust and refine the outcome domains, measures, and instruments for assessing program benefit. Additionally, future testing will further define the intervention’s target population by identifying characteristics of older adults with MCI or subjective memory decline who live alone and are most likely to benefit from the Home Alone program. Depending on how well the refined Home Alone intervention is received in Phase II, a subsequent randomized clinical trial will be necessary to evaluate its treatment effect.

## 5. Conclusion

Together, the findings of this pilot study demonstrate the early feasibility, acceptability, and utility of the Home Alone program. This novel, evidence‐based intervention shows promise for addressing a critical and growing unmet need: helping older adults with memory concerns who live alone to age in place with purpose, quality of life, and sustained engagement in meaningful activities.

## Funding

This work was supported by the National Institute on Aging at the National Institutes of Health (grant number 5R21AG080744‐02).

## Disclosure

The content of this manuscript is solely the responsibility of the authors and does not necessarily represent the official views of the National Institutes of Health.

## Conflicts of Interest

The authors declare no conflicts of interest.

## Supporting Information

Additional supporting information can be found online in the Supporting Information section.

## Supporting information


**Supporting Information 1** Item 1: General Session Agendas.

**Supporting Information 2** Item 2: Survey Measures.

**Supporting Information 3** Item 3: Semi‐Structured Interview Guide.

**Supporting Information 4** Item 4: Descriptive Statistics for Behavior, Health, and Cognition Measures.

**Supporting Information 5** Item 5: Finalized Codebook.

**Supporting Information 6** Item 6: Qualitative Analysis Flow Diagram.

**Supporting Information 7** Item 7: Demographics by Cognitive Status.

**Supporting Information 8** Item 8: Session Data Table.

## Data Availability

The data that support the findings of this study will be openly available in the National Archive of Computerized Data on Aging (NACDA), the National Institute on Aging/National Institutes of Health (NIA/NIH)‐funded repository, once Phase II of the study is complete. We will deposit the quantitative/close‐ended, item‐level, de‐identified data collected from questionnaires. The qualitative data from the semistructured interviews will not be made publicly available due to privacy restrictions.
