# Supplementary material for: Evaluating the Feasibility, Acceptability, and Utility of the Home Alone Intervention: A Mixed Methods Pilot Study
Source: J Aging Res. 2026 May 19;2026:4036735. doi: 10.1155/jare/4036735 (PMC13185217; doi:10.1155/jare/4036735)
Supplement: Supplementary file 1 — Supporting Information 1 Item 1: General Session Agendas. [file JARE-2026-4036735-s008.docx]

Supplementary Item 1. General Session Agendas

**Session 1 General Agenda – Participant Concerns and Home Safety**

State Agenda

Program and Session Expectations

- Ask questions to get an understanding participant’s social engagement and activity engagement quality of life, and mood as well as transportation issues/driving retirement, housing, health and function, safety, family dynamics and support, greater support network, current use of informal and formal supports and services and other potential areas of concern. Inquire about challenges as well as barriers contributing to, and timing of, any changes.
- Identify challenges and environmental aspects that may cause difficulties/home safety check.
- Identify goals for intervention
- Learn about SMART Goals goal-setting
- Homework:
  - Create one SMART goal for the week

**Session 2 General Agenda – Share Informal and Formal Supports and Services**

State Agenda

Session Expectations

Review SMART goal completion

- Share informal support options (family, neighbors, friends, volunteers, community organizations)
- Share formal support options (in-home health care, driving evaluations/training, occupational therapy evaluations, non-profit organizations, government organizations)
- Problem-solve to increase access and likely use of these supports
- Evaluate current and future issues for independent living, with an emphasis on encouraging realistic expectations
- Provide psychoeducation on SMART Goals goal-setting (if did not have time to do in Session 1)
- Introduce Mindfulness Based Stress Reduction strategies and relaxation exercises (deep breathing, 5-4-3-2-1 grounding technique, journaling)
  - - Can be used later as possible scheduled activity choices
- Homework:
  - Set one SMART goal

**Session 3 General Agenda - Behavioral Activation Introduction**

State Agenda

Session Expectations

Review Homework (SMART goal) completion

- Stress beginning to focus on Behavioral Activation
- Provide detailed explanation and rationale for use of behavioral activation. We will use **Activity and Engagement Education and Behavioral Activation and Intervention Rationale Handouts**
- Talk about Activity Monitoring **(will use Activity Rating Instructions handout and Daily Monitoring Form** **handout)**
- What do you do during a day:
  - How important is it?
  - How much do you enjoy it?
  - How does it make you feel?
- Complete an example **Daily Monitoring Form** of a day’s activities for day of session and day prior.
- Homework:
  - Participants complete **Daily Monitoring Form** for the week as best they can.

Talk about the importance of completing homework

**Session 4 General Agenda – Values and Activity Scheduling**

State Agenda

Session Expectations

Review Homework completion

- Talk about life areas, values, activities **(use Life Areas, Values, Activities structure handout)**
- Determine activities that reflect participant’s values in the 5 primary life areas – Relationships, Recreation/Interests, Mind, Body, & Spirituality, Daily Responsibilities, and Education/Career **(use Life Areas, Values, and Activities Inventory handout)**
- Identify your most important values for each Life Area
- Explore activities you like to do and determine activities to try based on your values **(use Life Areas, Values, and Activities Examples handout)**
- Complete 1-2 life area (s), strive for 6 values, 2 activities for each in **Life Areas, Values, and Activities Inventory handout**
- Create a list of 10 enjoyable and meaningful activities **(Activity Selection and Rating handout)**
- Homework:
  - Pick 4 activities and try to do them for the week.
  - Complete **Daily Monitoring Form**, and circle the 4 predetermined activities, if they are done
  - Could set SMART goal for completion of agreed upon number of the activities.

**Session 5 General Agenda – Intentional Scheduling**

State Agenda

Session Expectations

Review Homework completion

IMI Interest/Enjoyment questions

Introduce Activity Scheduling and the Weekly Activity Planning and Monitoring handout

Share log and we will talk about how to complete it:

- - Write in Life Areas, Values and Activities
  - Schedule desired activities.
  - Rate feasibility.
  - Tracking: After you have done an activity, rank the importance and enjoyment.

Homework:

- - Engage in 4 scheduled activities a day for the week
  - Complete **Weekly Activity Planning and Monitoring handout** logs for each day, if possible

**Session 6 General Agenda—Motivation/Troubleshooting**

- State Agenda

Session Expectations

Review Homework completion

IMI Interest/Enjoyment questions

- Review **Weekly Activity Planning and Monitoring log**
- Review reasoning behind and recommend engaging in activities, regardless of current mood or physical health.
- Discuss motivation tips/memory strategies **(using motivation tips handout)**
- Discuss importance of positive self-talk/self-encouragement
- Explore any successes and challenges encountered with intentional scheduling of positive activities
- Importance of commitment to sticking with activity scheduling, even when it’s a hard week.
- Preparing for obstacles and learn how to overcome them when goal-setting (for example, what might happen, what can I do if this does happen, who can help).
- Consider contracts, if need activities could benefit from others help **(Contracts handout)**
- Create new activity ideas list **(Activity Selection and Rating handout)**
- Homework:
  - Engage in 4-8 (or as many as the participant feels confident they can do) enjoyable activities a day
  - Complete **Weekly Activity Planning and Monitoring log** for each day, if possible

**Session 7 General Agenda – Review and Wrap Up**

State Agenda

Session Expectations

Review Homework completion

IMI Interest/Enjoyment questions

Anything that we haven’t covered

Celebrate successes/what went well with this program?

Any questions and/or concerns

Discuss moving forward/what comes next

Review purpose and importance of BA

- Create or review list of activities **(Activity Selection and Rating handout)**
- Homework:
  - Give homework for the week of completing a specified (by the participant) number of positive activities each day (using **Weekly Activity Planning and Monitoring log)**
