# Supplementary material for: Evaluating the Feasibility, Acceptability, and Utility of the Home Alone Intervention: A Mixed Methods Pilot Study
Source: J Aging Res. 2026 May 19;2026:4036735. doi: 10.1155/jare/4036735 (PMC13185217; doi:10.1155/jare/4036735)
Supplement: Supplementary file 2 — Supporting Information 2 Item 2: Survey Measures. [file JARE-2026-4036735-s006.docx]

Supplementary Item 2. Survey Measures

| Measure | Description & Citations | Administered |
| --- | --- | --- |
| Socio-Demographic Data | Collects data on age, education, marital status, gender, cognitive status, employment, income, and children. | Baseline |
| Blind/Telephone-MoCA | Adapted 5-10 minute version of the MoCA that evaluates attention, memory, language, abstraction, delayed recall, and orientation | Baseline, 1 Month, and 3 Months |
| Sensory Loss Questionnaire (Modified) | Items focus on hearing and vision adapted for the Home Alone Study. (8 items) (Doungkamol et al., 2001 and NHIS 2014 Adult Functioning and Disability Questionnaire) | Baseline, 1 Month, and 3 Months |
| Activities of Daily Living (ADLs) | Fifteen item measures asking about functional ability in personal care activities (e.g., bathing, dressing). (Katz et al., 1963). | Baseline, 1 Month, and 3 Months |
| Geriatric Depression Scale (Short-Form) | A 15-item self-report screening tool for depression in older adults (Sheikh et al., 1986). | Baseline, 1 Month, and 3 Months |
| DeJong Gierveld Loneliness Scale | A 6-item form evaluating emotional and social loneliness (De Jong Gierveld & Van Tilburg, 2006). | Baseline, 1 Month, and 3 Months |
| Lubben Social Network Scale-6 (LSNS-6) | A 6 item screening tool used for social isolation by measuring perceived social support from friends and family (Lubben et al., 2006). | Baseline, 1 Month, and 3 Months |
| Pleasant Events Schedule-AD | Measures frequency and enjoyment of pleasant activities tailored to Alzheimer’s and dementia populations (20 items). (Logsdon et al., 1997) | Baseline, 1 Month, and 3 Months |
| Utilization of Unpaid Social Support Care Resources (Modified) | A 9-item measure that captures use of informal unpaid support (family/friends). (Kasper et al., 2020) | Baseline, 1 Month, and 3 Months |
| Utilization of Paid Community-Based, Residential, or Other Services | Tracks use of formal services (e.g., home health care, residential care). (Concepts adapted from Amjad et al, 2018; Mittelman & Pierzchala, 2003): | Baseline, 1 Month, and 3 Months |
| US Health Interview Survey | Adapted items from the NHIS for general health and healthcare access measure looking at physical activity levels (12 items). (Sturman et al., 2005) | Baseline, 1 Month, and 3 Months |
| Behavioral Activation Scale | Modified measure for goal-directed activation and engagement in meaningful activities with 1 author created item (9 items). (Kanter et al., 2007) | Baseline, 1 Month, and 3 Months |
| Home Alone Treatment Review Checklist | Author created 10-item measure to assess perceptions of the intervention and likelihood to recommend to others. | 1 Month and 3 Months |
| Feasibility, Acceptability, and Appropriateness Measures | A psychometric measure evaluating implementation outcomes related to intervention delivery (12 items) (Weiner et al., 2017). | 1 Month and 3 Months |
