# Supplementary material for: Evaluating the Feasibility, Acceptability, and Utility of the Home Alone Intervention: A Mixed Methods Pilot Study
Source: J Aging Res. 2026 May 19;2026:4036735. doi: 10.1155/jare/4036735 (PMC13185217; doi:10.1155/jare/4036735)
Supplement: Supplementary file 3 — Supporting Information 3 Item 3: Semi‐Structured Interview Guide. [file JARE-2026-4036735-s005.docx]

Supplementary Item 3. Home Alone Semi-Structured Interview Guide - Phase I

Introduction:

Thank you so much for participating in our evaluation of the Home Alone program. To follow-up on your participation, I would like to ask some open-ended questions. For research purposes, our conversation will be recorded so it may be transcribed and studied at a later date. The purpose of this interview is to find out if you found Home Alone useful.

Opening question:

1. For the first question, are you still living alone at home?

Now, let’s talk about what you thought of the Home Alone program.

2. Was it useful to you? Why or why not?

3. What, if anything, about the program was the most helpful?

4. What, if anything, about the program was not helpful?

5. Was it helpful or not to discuss your experiences with your coach?

6. Did the program make it easier for you to talk about living alone?

Home Alone Target Outcomes:

We’re going to talk more specifically about how Home Alone worked for you now. {Prompt interviewee to tell you more, when needed}

7. Did Home Alone help you to do more fun activities?

8. Did Home Alone help you spend more time with other people?

9. Did Home Alone affect your mood and/or health?

10. Did Home Alone help you to learn about different kinds of supports and services?

- If yes, are you using any of these services/supports?

11. Did Home Alone help you feel more confident about safely living alone?

12. Did you make any changes around your house because of what you learned in the Home Alone program?

- If yes, what changes did you make?

Current Needs:

13. Do you have any concerns now about living alone?

a. Is anyone helping you with those concerns?

b. {If no one is helping with concerns, ask:}, Would you like to talk to your coach about that/those concern(s)?

{If answers yes:} We will have them contact you.

Questions Specific to Home Alone Delivery:

The next four questions are about the delivery of the Home Alone program:

14. Were the sessions too long, just right, or too short?

15. Were there too many, the right amount, or not enough sessions?

16. Did you like having your coach come to your home?

Just a few more questions and the interview will be done:

Improvements:

17. What, if anything, would you change or add to the Home Alone program to make it better?

Final question:

18. Thank you for helping us learn more about your experiences with Home Alone. Is there anything else you would like to say or add?

Thank you so much for joining us for the Home Alone interview! We truly appreciate you sharing your experiences. We will put $25 as a thank you on your ClinCard as a token of our appreciation.

After the study, we will share a summary of what we have found. In the meantime, please do not hesitate to reach out if you have any questions or concerns.
