# Supplementary material for: Evaluating the Feasibility, Acceptability, and Utility of the Home Alone Intervention: A Mixed Methods Pilot Study
Source: J Aging Res. 2026 May 19;2026:4036735. doi: 10.1155/jare/4036735 (PMC13185217; doi:10.1155/jare/4036735)
Supplement: Supplementary file 4 — Supporting Information 4 Item 4: Descriptive Statistics for Behavior, Health, and Cognition Measures. [file JARE-2026-4036735-s004.docx]

Supplementary Item 4. Descriptive Statistics for Behavior, Health, and Cognition Measures

|  | **T0** | **T1** | **T2** |
| --- | --- | --- | --- |
|  | N=15 | N=15 | N=15 |
| **Mean Sum Scores** | **Mean (SD)** | **Mean**  **(SD)** | **Mean**  **(SD)** |
| Geriatric Depression Scale | 4.10 (3.48) | 3.50  (3.46) | 3.60  (3.50) |
| Activities of Daily Living | 2.20 (2.86) | 2.20  (2.20) | 2.50  (2.90) |
| Social Support Usage | 0.87 (1.60) | 1.07  (1.50) | 1.33  (1.50) |
| Paid Support Service Usage | 0.93  (1.00) | 1.33  (1.20) | 1.10  (1.33) |
| Interest in Using a Paid Support Service | 1.40 (2.06) | 1.20  (1.80) | 1.30  (2.50) |
| Behavioral Activation Scale | 29.90 (8.50) | 34.50  (7.90) | 32.60  (8.70) |
| Lubben Social Network Scale | 16.50 (6.20) | 19.90  (6.20) | 14.50  (5.50) |
| De Jong Gierveld Scale (Short) | 2.33  (1.63) | 3.47  (0.83) | 3.40  (0.83) |
| Pleasant Event Schedule: Frequency | 29.53  (2.56) | 29.79  (3.53) | 28.27  (5.60) |
| Pleasant Event Schedule: Enjoys Now | 15.13  (2.13) | 16.0  (2.51) | 17.40  (1.59) |
| Physical Activity Engagement | 3.10  (1.40) | 3.80  (3.30) | 2.80  (1.70) |
| Hearing Impairment: Social Frustration | 3.87  (0.99) | 4.00  (1.07) | 3.93  (1.03) |
| Hearing Impairment: Safety | 4.67  (0.62) | 4.73  (0.59) | 4.67  (0.49) |

| Vision Impairment: Social Frustration | 4.27  (0.96) | 4.40  (0.83) | 4.20  (1.01) |
| --- | --- | --- | --- |
| Vision Impairment: Safety | 4.40  (0.91) | 4.33  (1.05) | 4.13  (1.06) |
| B/T-MoCA | 18.93  (2.79) | 18.40  (3.81) | 17.67  (3.50) |
