# Supplementary material for: Evaluating the Feasibility, Acceptability, and Utility of the Home Alone Intervention: A Mixed Methods Pilot Study
Source: J Aging Res. 2026 May 19;2026:4036735. doi: 10.1155/jare/4036735 (PMC13185217; doi:10.1155/jare/4036735)
Supplement: Supplementary file 5 — Supporting Information 5 Item 5: Finalized Codebook. [file JARE-2026-4036735-s003.docx]

Supplementary Item 5. Finalized Codebook

**Section 1: Impacts (both positive and negative captured in the codes)**

| NVivo Code Name | Description |
| --- | --- |
| 1.1 Socialization |  |
| Impacts Socialization | Improves/did not improve socialization with others; refers to when participants talk about spending time with others, awareness of socialization is assumed |
| Awareness of Socialization | Increased/did not increase awareness of socialization opportunities; refers to knowing about not being reclusive; participants know about opportunities but don’t implement them |
| Promotes Conversation About Living Alone | Promotes or does not promote conversation about living alone with friends, family, healthcare professionals |

| NVivo Code Name | Description |
| --- | --- |
| 1.2 Well-Being |  |
| Impacts Confidence | Program built/did not build confidence in living alone; pertains to an internalized feeling confident in living alone, program did/did not equip participant with confidence to manage future concerns (not confidence in talking to others, which falls under either Promotes Conversation About Living Alone or Self-Advocacy, depending on context) |
| Affirmation | Program provides/doesn't provide positive affirmation of participants; affirmed for certain lifestyle choices and decisions and “doing the right thing” |
| Self-Advocacy | Program fosters/does not foster self-advocacy; ability to talk others to meet needs or find/start resources/services; no impact on self- advocacy; participant comments around proactiveness, standing up, seeking assistance |
| Impacts Mood/Health | How program improves/did not improve mood/health; comments on how participants felt as a result of program, any health discussions (physical health, doctor appointment, mental well-being, coaching sessions brightened mood, appreciation or gratitude that program exists) |
| Purpose | Participating in the program gave/did not give them a sense of purpose |

| NVivo Code Name | Description |
| --- | --- |
| 1.3 Values Identification | Participants identifying values that are important to them; mentioning “value assessments” |

| NVivo Code Name | Description |
| --- | --- |
| 1.4 Meaningful Activity Engagement | Did/did not engage in meaningful activities (new, same, or decrease) or focused on activity prioritization; participants talk about the program encouraging or impacting activities (picking up new hobbies, doing enough, determining the personal right amount of activities) awareness of meaningful activity awareness is assumed |
| 1.5 Meaningful Activity Awareness | Program did or did not increase awareness of, or participants mention discussing activities that are meaningful. Participants mention thinking of fun activities, but not yet engaging in them |

| NVivo Code Name | Description |
| --- | --- |
| 1.5 Strategies and Resources |  |
| Awareness of external services/resources | Program increased/did not impact awareness or use of services, supports, resources; keeping information for use in the future or wishing program included more specific information on resources or service or support |
| Utilization of new services/external resources | Utilized new services/supports/resources mentioned in program or did not use new services/supports/resources or was already, awareness of external services/resources is assumed using or irrelevant to them |
| Awareness of daily living and motivational strategies and memory aids | Practical tips and tricks/daily living strategies, promotion of organization; establishing routine, cataloging of information already known, building awareness of strategies/aids; goal-setting (do not use this code if referring to external services and supports) |
| Implementation of daily living and motivational strategies and memory aids | Implementation of practical tips and tricks/daily living strategies, organizational strategies; established routine, utilized goal-setting (do not use this code if referring to external services and supports), awareness of daily living strategies is assumed |

| NVivo Code Name | Description |
| --- | --- |
| 1.6 Home Safety |  |
| Awareness of home safety/home safety modifications | Program increased/did not increase awareness of home safety risks, home safety modifications;(cords, bad lighting, throw rugs, tripping hazards, locking doors); (grab bars, handheld showerheads, night lights, stair railings, decluttering; organization) |
| Implementation of home safety modifications | Implemented home safety modifications or did not implement home safety modifications, or they were not needed, awareness of home safety is assumed |

**Section 2. Intervention Structure and Delivery**

| NVivo Code Name | Description |
| --- | --- |
| 2.1 General Program | Comments about general structure/design of intervention program; likes or dislikes of broad intervention structure, overarching design, virtual vs in person engagement, general program comments |

| NVivo Code Name | Description |
| --- | --- |
| 2.2 Coaching |  |
| Personalization | How coaching sessions were personalized to meet participant-specific needs |
| Engagement with coach | Listening, one-on-one conversations, dialogue, reflection of topics; feedback; rapport, also talking about future ad-hoc sessions with coach |
| Accountability | Comments about feeling accountable to coach |

| NVivo Code Name | Description |
| --- | --- |
| 2.3 Workbook/  workbook activity logs | Enjoyed working through the workbook; thought it was valuable; liked the content, thought it was too much work, didn't think it was relevant, didn't like the content; anything pertaining to the activity logs that were completed outside of session (content about workbook can go in Section 1 codes) |

**Section 3. Recommendations and Modifications**

| NVivo Code Name | Description |
| --- | --- |
| 3.1 Future changes | Comments around how the intervention can be modified in the future (exclusions, inclusions, content ideas, wearables). Does not apply to comments on surveys. |
| 3.2 Program "Fit" | Comments about whom the program might work best/least for |

**Section 4. Good Quotes**

| NVivo Code Name | Description |
| --- | --- |
| 4.1 Good Quotes | Any quotes that would be good for the paper |

**Section 5. Future Concern Examples**

| NVivo Code Name | Description |
| --- | --- |
| 5.1 Future Concerns | Comments with examples of concerns for their future |
