# Supplementary material for: Evaluating the Feasibility, Acceptability, and Utility of the Home Alone Intervention: A Mixed Methods Pilot Study
Source: J Aging Res. 2026 May 19;2026:4036735. doi: 10.1155/jare/4036735 (PMC13185217; doi:10.1155/jare/4036735)
Supplement: Supplementary file 6 — Supporting Information 6 Item 6: Qualitative Analysis Flow Diagram. [file JARE-2026-4036735-s002.docx]

Supplementary Item 6. Qualitative Analysis Flow


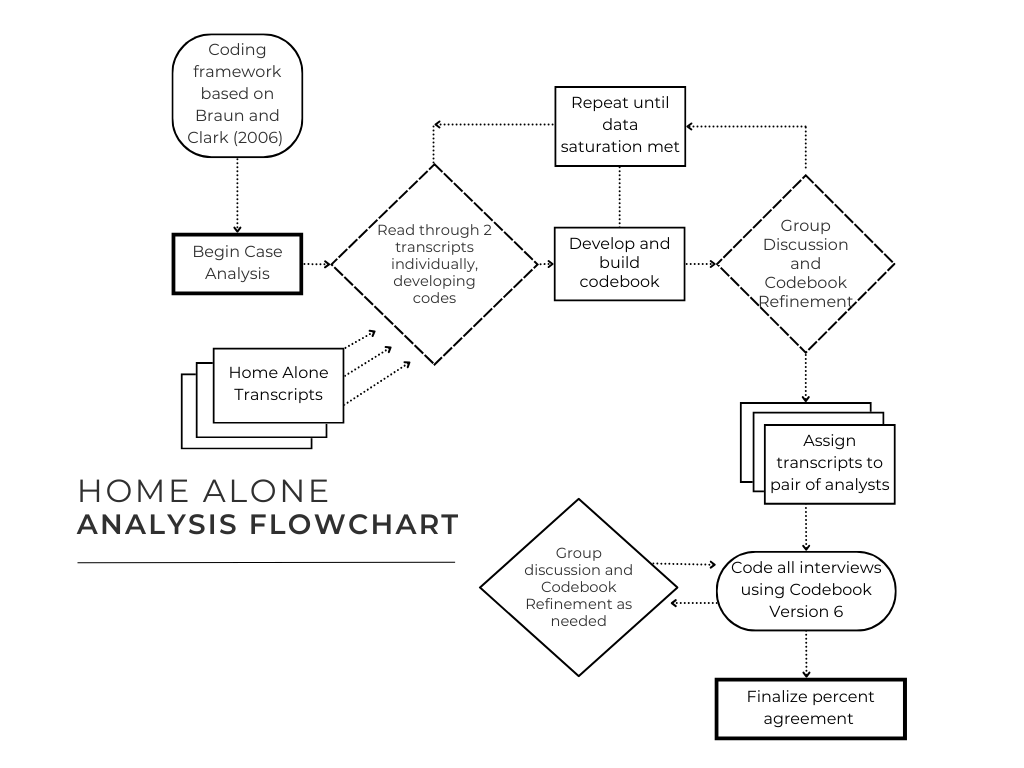
Note. Adapted from Damschroder & Lowery (2013) and Assarroudi et al. (2018).
