# Supplementary material for: Evaluating the Feasibility, Acceptability, and Utility of the Home Alone Intervention: A Mixed Methods Pilot Study
Source: J Aging Res. 2026 May 19;2026:4036735. doi: 10.1155/jare/4036735 (PMC13185217; doi:10.1155/jare/4036735)
Supplement: Supplementary file 7 — Supporting Information 7 Item 7: Demographics by Cognitive Status. [file JARE-2026-4036735-s001.docx]

Supplementary Item 7. Demographics by Cognitive Status

|  | MCI/ADRD Diagnosis  (n = 5) | T-MoCA Score in MCI range  (n = 5) | Subjective Cognitive Decline  (n = 5) |
| --- | --- | --- | --- |
|  | % | % | % |
| Male | 40 | 40 | 0 |
| White | 80 | 80 | 100 |
| Married | 0 | 0 | 0 |
| Retired | 80 | 80 | 80 |
| Post High School Education | 100 | 100 | 100 |
| Above $40,000 a year | 60 | 40 | 60 |
| Age | 73.8 (Mean) | 73.8 (Mean) | 72.4 (Mean) |
