# Supplementary material for: Evaluating the Feasibility, Acceptability, and Utility of the Home Alone Intervention: A Mixed Methods Pilot Study
Source: J Aging Res. 2026 May 19;2026:4036735. doi: 10.1155/jare/4036735 (PMC13185217; doi:10.1155/jare/4036735)
Supplement: Supplementary file 8 — Supporting Information 8 Item 8: Session Data Table. [file JARE-2026-4036735-s007.docx]

Supplementary Item 8. Session Data

| *Intervention* | Total Number of Sessions | 42 |
| --- | --- | --- |
|  | Average Session Length (Minutes) | 60.38, SD = 10.06, Range 30-90 |
| *Ad hoc* | Participants | 14 (93%) |
|  | 35 Ad Hoc Communications | · 32 Email Communications  · 3 Check-in Calls (study-task related, e.g. requests for survey completion or sharing resources following survey completion) |
|  | Average Ad Hoc Communication Length (Minutes) | Email: 16.56, SD = 10.51, Range 5-30  Check-in Calls: 11.67, SD = 7.64, Range 5-20 |
